# Evaluation of Subfoveal Choroidal Thickness in Uncomplicated and Hypertensive Disorders of Pregnancy Using Spectral-Domain Optical Coherence Tomography and Its Postpartum Correlation

**DOI:** 10.7759/cureus.89709

**Published:** 2025-08-09

**Authors:** Richa Nyodu, Vidhya Verma, Samendra Karkhur, Priti Singh, Saroj Gupta

**Affiliations:** 1 Ophthalmology, Shroff Eye Centre, New Delhi, IND; 2 Ophthalmology, All India Institute of Medical Sciences, Bhopal, Bhopal, IND

**Keywords:** choroidal vasculature, enhanced depth imaging, hypertensive disorders of pregnancy, ocular biomarkers, postpartum changes, spectral domain oct, subfoveal choroidal thickness, uncomplicated pregnancy

## Abstract

Purpose

This study aimed to evaluate subfoveal choroidal thickness (SFCT) in women with uncomplicated pregnancy and those with hypertensive disorders of pregnancy (HDP), using spectral-domain optical coherence tomography (SD-OCT), and to assess changes in SFCT during the postpartum period.

Methods

The study was jointly carried out in the Departments of Ophthalmology and Obstetrics & Gynaecology at All India Institute of Medical Sciences, Bhopal, a tertiary care referral centre in central India, over a period of 18 months, from March 2021 to September 2022. A hospital-based cross-sectional study was conducted on pregnant women in their third trimester, including those with HDP and those with uncomplicated pregnancies. All participants underwent routine antenatal work-up, comprehensive ocular examination, and enhanced depth imaging optical coherence tomography (EDI-OCT) to measure SFCT. Follow-up assessments were performed at six weeks postpartum to evaluate postnatal changes in choroidal thickness.

Results

A total of 96 eyes from 48 pregnant women were evaluated. An overall increase in SFCT was observed in the postpartum period; however, this increase was not statistically significant, possibly due to the small sample size and loss to follow-up postpartum. In addition, the mean SFCT was higher in the uncomplicated pregnancy group compared to those with HDP, although this difference was also statistically insignificant. Despite limited statistical power, these findings suggest that HDP may influence choroidal thickness differently compared to normotensive pregnancies.

Conclusion

This study highlights the potential role of SFCT as a non-invasive biomarker for early identification and risk stratification of hypertensive disorders in pregnancy. Although the results did not reach statistical significance, the observed trends support the hypothesis that choroidal vascular changes may reflect underlying systemic vascular dysregulation in HDP. Future research with larger sample sizes and longer follow-up is warranted to validate these findings and to explore the utility of mean-adjusted choroidal thickness as a predictive marker for HDP severity and progression.

## Introduction

The choroid, the posterior part of the uvea, consists of five layers: the outer pigment epithelium, Haller’s layer, Sattler’s layer, the choriocapillaris, and Bruch’s membrane. It is richly vascularized by the posterior ciliary arteries and plays a key role in ocular nutrition and regulation of blood flow [1]. Choroidal thickness is influenced by several physiological factors, including age, sex, axial length, refractive error, circadian rhythm, and hormonal fluctuations, particularly across the menstrual cycle. Estrogen receptor mRNA has been detected in choroidal cells, suggesting hormonal modulation [2].

Pregnancy induces systemic hemodynamic and hormonal changes that enhance blood volume, cardiac output, and vascular permeability. These changes can alter ocular circulation and have been associated with central serous chorioretinopathy (CSCR), with pregnancy increasing CSCR risk by up to seven times [3]. Hypertensive disorders of pregnancy (HDP), such as preeclampsia and eclampsia, complicate approximately 2-8% of pregnancies and are major contributors to maternal morbidity and mortality [4]. These conditions are characterized by endothelial dysfunction, increased vascular resistance, and ocular manifestations such as retinal edema, haemorrhages, detachment, and chorioretinal atrophy [5].

Visual symptoms occur in around 25% of preeclamptic and 50% of eclamptic women. Findings include blurred vision, photopsia, scotomata, and even cortical blindness. Ocular changes stem from choroidal ischemia, breakdown of the blood-retina barrier, and retinal pigment epithelium (RPE) dysfunction, often resolving postpartum but occasionally causing lasting damage [6].

Earlier imaging modalities like fluorescein angiography, indocyanine green angiography, and ultrasound were limited by safety concerns or insufficient resolution, especially during pregnancy. Spectral-domain optical coherence tomography (SD-OCT) with enhanced depth imaging now enables non-invasive, high-resolution, in vivo measurement of subfoveal choroidal thickness (SFCT) [7].

However, existing literature presents conflicting findings on SFCT in preeclampsia; some studies report increased thickness due to vascular hyperpermeability, while others attribute reduced SFCT to vasospasm and decreased perfusion, highlighting the need for further study.

## Materials and methods

Study design and setting

This hospital-based, cross-sectional observational study was conducted after obtaining ethical clearance from the Institutional Ethics Committee of All India Institute of Medical Sciences (AIIMS), Bhopal (Ref. 2020/PG/July/28), adhering to the tenets of the Declaration of Helsinki (1964). Written informed consent was obtained from all participants prior to enrolment. The study was jointly carried out in the Departments of Ophthalmology and Obstetrics & Gynaecology at All India Institute of Medical Sciences, Bhopal, a tertiary care referral centre in central India, over a period of 18 months, from March 2021 to September 2022.

Study participants

Pregnant women beyond 20 weeks of gestation who attended the outpatient Department of Obstetrics and Gynaecology and fulfilled the inclusion criteria were enrolled after providing informed consent. The inclusion criteria for the case group (HDP) consisted of pregnant women beyond 20 weeks of gestation diagnosed with HDP and aged between 18 and 45 years. Patients diagnosed with HDP were managed according to standard obstetric protocols, which included initiation of antihypertensive medications when clinically indicated. All relevant clinical and ophthalmic data, including blood pressure and OCT measurements, were recorded at the time of diagnosis of HDP (antenatal) and during the first postpartum follow-up visit. The control group (Uncomplicated Pregnancy) included pregnant women beyond 20 weeks of gestation without systemic illnesses and having systolic blood pressure less than 140 mmHg and diastolic pressure less than 90 mmHg.

Pregnant females with systemic conditions such as cardiac disease, thyroid disorders, and inflammatory diseases with vascular involvement, including rheumatoid arthritis or Behcet disease, as well as those diagnosed with eclampsia, were excluded. Ocular exclusion criteria comprised individuals with age-related macular degeneration, diabetic retinopathy, glaucoma, or significant refractive errors greater than 2 diopters of cylindrical or 3 diopters of spherical correction. Additional ocular exclusions included a history of previous intraocular surgery or laser therapy, presence of cataract, corneal opacity, or any media opacity that hampered the signal strength of the enhanced depth imaging-optical coherence tomography (EDI-OCT). Retinal vascular occlusions or other retinal pathologies altering the anatomical integrity of the retina were also excluded. Furthermore, smokers, drug abusers, those unwilling to provide consent, and patients with incomplete medical records were not considered. In the control group, women who later developed hypertensive disorders during the course of their pregnancy were also excluded.

Sample size estimation

Based on the study by Sharudin SN et al. [8], which reported mean subfoveal choroidal thickness (SFCT) values of 370.7 ± 23.8 µm in hypertensive pregnancies and 344.5 ± 30.8 µm in uncomplicated pregnancies, the effect size was calculated as 0.952 using G*Power 3.1.9.7 (Heinrich Heine University of Düsseldorf (HHU), Germany). A minimum sample size of 38 participants (19 per group) was estimated. However, to enhance statistical power, all eligible participants during the 18-month study duration were included.

Study variables and clinical examinations

All participants were evaluated for demographic characteristics such as age and underwent a detailed medical and surgical history assessment. Blood pressure measurements were recorded at both the initial and postpartum visits. A comprehensive ophthalmic examination was performed for each participant, which included assessment of visual acuity, intraocular pressure (IOP), and slit-lamp examination. SD-OCT using a 6 mm enhanced depth imaging (EDI) scan was performed for choroidal thickness measurement. Routine laboratory investigations conducted for all participants included complete blood count (CBC), erythrocyte sedimentation rate (ESR), random blood sugar (RBS), hepatitis B surface antigen (HBsAg), anti-HCV, HIV types I and II, urine microscopy, chest X-ray, renal and liver function tests (RFT, LFT), electrocardiography (ECG), fasting and postprandial blood sugar (FBS, PPBS), glycated hemoglobin (HbA1c), and lipid profile. These investigations were carried out in accordance with clinical protocols as advised by the Department of Obstetrics and Gynaecology.

Clinical examination variables were documented by an academic junior resident (RN) under the supervision of a vitreoretinal surgeon with experience in assessing over 1,000 OCT scans. Choroidal thickness measurements using HD-OCT were recorded twice, once during the antenatal period and again postpartum. Comparative analysis was conducted between women with HDP and those with uncomplicated pregnancies, all beyond 20 weeks of gestation.

OCT imaging and analysis

SD-OCT was performed using HD OCT-Cirrus (Carl Zeiss Meditec, Dublin, CA, USA), equipped with an optical source of a superluminescent diode (SLD) at 840 nm. The device operated at scan speeds ranging from 27,000 to 68,000 A-scans per second and had an axial resolution of 5 µm and a transverse resolution of 15 µm in tissue. The EDI mode provided by the manufacturer was used to obtain enhanced choroidal images. Transfoveal horizontal and vertical line scans, each composed of 100 averaged B-scans, were obtained for SFCT measurements. The built-in calliper of the HD Cirrus OCT 500 was used to measure SFCT at the subfoveal point, extending from the outer border of the hyper-reflective retinal pigment epithelium (RPE) line to the sclerochoroidal interface. The 6 mm macular map scan was also evaluated for additional OCT findings such as pigment epithelial detachment (PED), subretinal fluid (SRF), and disruption of the inner segment-outer segment (IS-OS) junction. These findings were recorded during each visit for all participants.

Data collection and analysis

All clinical and imaging data were entered into a computer-based spreadsheet. Continuous variables were summarized using mean and standard deviation, while categorical variables were expressed as proportions and percentages. The normality of data distribution was assessed using the Kolmogorov-Smirnov test. Depending on the data distribution, either the Mann-Whitney U test or the independent t-test was used to assess statistically significant differences between the groups. All statistical analyses were performed using IBM SPSS Statistics for Windows, version 26.0 (IBM Corp., Armonk, NY, USA).

## Results

The present study was conducted on a total of 48 pregnant women attending the antenatal clinic in the Department of Obstetrics and Gynaecology at AIIMS.

The demographic characteristics, clinical parameters, and ocular findings were systematically recorded and analyzed. The results are presented below through comprehensive tables and graphical representations, summarizing the key observations and comparisons between the hypertensive and normotensive groups.

Table 1 presents the mean values and standard deviations for key sociodemographic and clinical parameters of the study participants. The average age of the participants was 28.46 ± 4.69 years.

**Table 1:** Summary statistics of continuous variables (n = 48)

Characteristic	Mean (SD)
Age of the participant (years)	28.46 (± 4.69)
Left eye	
• Antenatal SFCT (µm)	319.79 (± 36.10)
• Postnatal SFCT (µm)	322.38 (± 37.62)
Right eye	
• Antenatal SFCT (µm)	319.10 (± 34.25)
• Postnatal SFCT (µm)	324.50 (± 39.14)
Gestational age (in days)	238.30 (± 28.54)
Systolic BP (antenatal) (mmHg)	132.35 (± 19.86)
Diastolic BP (antenatal) (mmHg)	85.58 (± 14.74)
Mean arterial pressure (antenatal) (mmHg)	101.42 (± 15.35)
Intraocular pressure (IOP)	
• Right eye (mmHg)	15.42 (± 2.40)
• Left eye (mmHg)	15.67 (± 2.58)
Pulse rate (beats/min)	91.00 (± 13.97)

The mean subfoveal choroidal thickness (SFCT) measured during the antenatal period was 319.79 ± 36.10 µm in the left eye and 319.10 ± 34.25 µm in the right eye. Postnatal SFCT measurements showed a slight increase, with the left eye averaging 322.38 ± 37.62 µm and the right eye 324.50 ± 39.14 µm.

The mean gestational age at the time of evaluation was 238.30 ± 28.54 days. The average systolic and diastolic blood pressures recorded during the antenatal period were 132.35 ± 19.86 mmHg and 85.58 ± 14.74 mmHg, respectively.

IOP measurements revealed a mean of 15.42 ± 2.40 mmHg in the right eye and 15.67 ± 2.58 mmHg in the left eye. These values reflect the baseline physiological and ocular characteristics of the study population.

The majority of participants were over 30 years of age (43.75%), while the smallest proportion belonged to the 18-25-year age group (27.08%). Most participants were primigravida (47.92%), whereas only one participant each was gravida 4 and gravida 5, as shown in Table 2.

**Table 2:** Categorization of participants according to their gravida status at time of admission (n = 48)

Gravida status	N (%)
Primi	23 (47.92%)
G2	16 (33.33%)
G3	7 (14.58%)
G4	1 (2.08%)
G5	1 (2.08%)

Most participants (75%) had a gestational age of 37 weeks or less at the time of evaluation. Regarding urinary albumin levels, 43.75% had trace albumin, while 39.58% had no detectable albumin.

Among the study participants, 37.5% were diagnosed with gestational hypertension, 4.17% with pre-eclampsia, and 8.33% with severe pre-eclampsia.

SFCT was assessed in both eyes before and after delivery. On comparing the antenatal and postnatal SFCT values for the left and right eyes, no statistically significant differences were observed in the mean SFCT measurements in Table 3.

**Table 3:** Changes in SFCT pre- and post-delivery in both eyes (n = 48) Unit: subfoveal choroidal thickness (SFCT) in micrometers (µm). Statistical test: paired t-test.

Eye	Parameter	Mean (SD)	t-value	p-value
Right eye	Antenatal SFCT	319.10 (± 34.25)	-0.719	0.47
	Postnatal SFCT	324.50 (± 39.14)		
Left eye	Antenatal SFCT	319.79 (± 36.10)	-0.343	0.73
	Postnatal SFCT	322.38 (± 37.62)		

The mean postnatal SFCT in both right and left eyes was compared between hypertensive and normotensive participants. No statistically significant difference was observed between the groups in either eye.

An overall increase in postnatal SFCT was noted in both the HDR and uncomplicated pregnancy groups. However, this change was not statistically significant. The observed trend suggests a possible variation in SFCT during the postpartum period, which may become clearer with a larger sample size and extended follow-up (Tables 4, 5)

**Table 4:** Difference in antenatal SFCT in both eyes based on hypertensive status as diagnosed during antenatal care (n = 48) Unit: subfoveal choroidal thickness (SFCT) in micrometers (µm). Statistical test: independent t-test.

Eye	Parameter (antenatal SFCT)	Mean (SD)	t-value	p-value
Right eye	Hypertensive (n = 24)	313.33 (± 30.02)	-1.17	0.25
	Normotensive (n = 24)	324.87 (± 37.77)		
Left eye	Hypertensive (n = 24)	316.08 (± 27.94)	-0.71	0.48
	Normotensive (n = 24)	323.50 (± 43.04)		

**Table 5:** Difference in postnatal SFCT in both eyes based on the hypertensive status as diagnosed during postnatal care (n = 48) Unit: subfoveal choroidal thickness (SFCT) in micrometers (µm). Statistical test: independent t-test.

Eye	Parameter (postnatal SFCT)	Mean (SD)	t-value	p-value
Right eye	Hypertensive (n = 24)	322.63 (± 39.74)	-0.33	0.74
	Normotensive (n = 24)	326.38 (± 39.29)		
Left eye	Hypertensive (n = 24)	322.00 (± 37.49)	-0.06	0.95
	Normotensive (n = 24)	322.75 (± 38.55)		

The increase in SFCT was more pronounced in the uncomplicated pregnancy group compared to those with HDR, but this difference also did not reach statistical significance. Mann-Whitney U test or t-test was applied to analyze changes in SFCT before and after delivery in both eyes, as well as to compare antenatal and postnatal SFCT values between hypertensive and normotensive participants. No statistically significant differences were observed in the mean SFCT of either the right or left eye across any of the comparisons.

## Discussion

The present study was conducted in the Department of Ophthalmology in collaboration with the antenatal clinics of the Department of Obstetrics and Gynaecology at a tertiary health centre of central India over a duration of 18 months. It aimed to evaluate changes in SFCT among antenatal and postnatal women, comparing those with uncomplicated pregnancies to those with HDP, using SD-OCT.

From a sociodemographic perspective, the majority of study participants were over 30 years of age. A significant proportion, approximately 47.92%, were primigravida and had been admitted to the antenatal care ward before 37 weeks of gestation. This trend aligns with recent national demographic data, which indicates a rising age of first pregnancy in Indian women, reflecting broader global reproductive trends. Only one earlier study, by Liu et al., has shown maternal age to be statistically significant in influencing choroidal thickness [9]. Other studies, such as those by Takahashi et al., found no such association [10]. Furthermore, none of the previous literature appears to have examined the impact of gravidity as an independent variable on SFCT, making this an unexplored area that warrants further attention.

In the current study, while a postnatal increase in SFCT was observed in both hypertensive and normotensive participants, this increase did not reach statistical significance. These findings are partly supported and partly contradicted by prior literature. Alizadeh et al. conducted a longitudinal assessment across pregnancy trimesters and into the postpartum period, documenting a progressive increase in SFCT from the first to third trimester, followed by a significant reduction postnatally [11]. Their findings suggest that pregnancy-related choroidal thickening may reverse after delivery, likely due to the normalisation of systemic hemodynamics and hormonal status.

A cross-sectional study found that healthy third-trimester pregnant women had increased choroidal thickness in specific macular regions compared to non-pregnant controls [12]. Desideri et al. similarly reported higher choroidal thickness in pregnant participants compared to age-matched non-pregnant controls, confirming pregnancy-induced ocular vascular changes [13]. Other studies corroborated these findings by showing significant choroidal thickening during pregnancy, which subsequently reduced after delivery. Despite methodological differences, most of these studies highlight that pregnancy impacts SFCT, although the extent and sustainability of these changes postpartum remain unclear.

Regarding hypertensive status, the mean SFCT in normotensive women was higher than in those with HDP, although the difference was not statistically significant in either eye or at any time point. This result contrasts with findings from several prominent studies. For instance, Fukui et al. reported significantly thicker SFCT in pre-eclamptic women than in normotensive pregnant or non-pregnant controls [14]. Sayin et al. also found increased SFCT in normotensive pregnant women compared to both pre-eclamptic and non-pregnant women, suggesting that physiological changes in pregnancy might be dampened in pathological states like pre-eclampsia [15]. On the other hand, Atas et al. found no significant difference in SFCT between pre-eclamptic and normotensive pregnant women, aligning with the present findings [16]. This discrepancy across studies could be attributed to variations in study design, population characteristics, gestational age at measurement, severity of HDP, and timing of OCT assessments.

Several physiological mechanisms may account for the variations observed in SFCT during pregnancy. Hormonal changes, increased vascular permeability, fluid retention, systemic vasodilation, decreased colloidal osmotic pressure, and prostaglandin-mediated vascular changes all potentially contribute to increased choroidal thickness during pregnancy [17]. These factors may operate differently in normotensive versus hypertensive pregnancies, adding to the complexity of interpretation.

The heterogeneity in findings across studies emphasises the need for multicentric, prospective research encompassing diverse populations with standardized imaging protocols and longitudinal follow-up. Differences in choroidal thickness may also stem from unaccounted confounding factors such as axial length, baseline central choroidal thickness (CCT), corneal curvature and biomechanics, and diurnal variations, all of which influence SFCT [1]. While SFCT measurements in this study were performed during OPD hours (9 AM to 5 PM) to reduce diurnal variation bias, exact matching of time points was not ensured, and this remains a limitation.

In addition, the role of antihypertensive medications was not uniformly controlled. The types, dosages, and timing of initiation of antihypertensives were heterogeneous and could have influenced ocular vascular status. Without precise data on medication usage and hemodynamic profiles, drawing definitive conclusions about the effect of hypertension or its management on SFCT remains challenging.

Interestingly, this study also documented retinal changes in individual cases, underscoring clinical relevance. One participant with pre-eclampsia showed a small amount of subretinal fluid, which resolved spontaneously after delivery. Another pre-eclamptic patient developed CSCR, which also resolved postpartum. These cases reinforce the hypothesis that increased choroidal permeability during pregnancy can lead to serous retinal detachment or CSC. Both cases showed increased SFCT during the antenatal period, followed by spontaneous clinical and anatomical resolution postpartum, with a corresponding reduction in choroidal thickness. These individual findings support the hypothesis that increased choroidal vascular permeability during pregnancy, particularly in the context of HDP, plays a contributory role in the development of CSC or related retinal changes. Prior research supports this: CSC in pregnancy is thought to be triggered by heightened choroidal permeability, hormonal influences, and autonomic dysregulation [18]. A case-control study reported an odds ratio of 7.1 for developing CSC in pregnant women compared to non-pregnant controls [19].

The relationship between systemic blood pressure and SFCT also remains inconclusive. Ahn et al. reported increased SFCT in patients with serous retinal fluid, which decreased after blood pressure normalisation [20]. Systolic blood pressure (SBP) and choroidal thickness (CT) exhibit significant diurnal variations; however, the relationship between them is not directly proportional. Iwase et al. demonstrated that, due to the choroid’s limited autoregulatory capacity, its blood flow is more influenced by systemic circulatory factors such as blood pressure, and variations in SBP do not consistently produce corresponding changes in choroidal thickness [21]. However, the extent to which rapid changes in blood pressure, such as those seen in pre-eclampsia, impact choroidal thickness is not yet fully understood. The proposed pathophysiology suggests that uteroplacental insufficiency, endothelial dysfunction, and impaired autoregulation in HDP could all contribute to altered choroidal structure and function.

Taken together, the findings from this study suggest a trend toward increased SFCT in pregnancy, particularly in normotensive women, with a tendency to reduce postpartum. The prospective design, standardized imaging protocol, and inclusion of postpartum follow-up strengthen the reliability of the observations. In addition, documentation of individual cases with retinal changes adds clinical relevance to the findings. However, due to the small sample size, lack of medication control, and absence of long-term follow-up, these trends could not be confirmed as statistically significant. Importantly, the observed trends mirror previously documented physiological hypotheses and highlight areas for further investigation.

Future studies should incorporate a larger sample size, consider confounding variables like axial length and systemic vascular parameters, and standardize OCT measurement protocols across gestational timelines. In addition, correlating SFCT with placental perfusion indices, blood pressure trends, and retinal vascular changes could further elucidate the interplay between ocular and systemic vascular health during pregnancy.

While the current study did not find statistically significant changes in SFCT between hypertensive and normotensive pregnant women or between antenatal and postnatal periods, subtle trends were observed that align with the physiological expectations and findings from other studies. These results suggest a role for SFCT as a potential non-invasive marker for pregnancy-induced vascular changes and warrant further investigation with a robust methodological design.

## Conclusions

No significant difference in SFCT was observed between normotensive and hypertensive pregnancies, although normotensive women showed slightly higher mean values. Postnatal SFCT increased in both groups but remained statistically insignificant. While SFCT may not serve as a predictive marker for HDP, subtle choroidal changes suggest differential vascular response. 
